# From factory floors to dream doors: a systematic review of sleep interventions in industrial workers

**DOI:** 10.1186/s12889-026-26239-1

**Published:** 2026-01-14

**Authors:** Sasha Javanmardi, Kristina Klier, Ludwig Rappelt, Daniel Niederer

**Affiliations:** 1 KNIPEX-Werk C. Gustav Putsch KG, Wuppertal, Germany; 2 Department of Computer Science, University of the Bundeswehr Munich, Neubiberg, Germany; 3 Department of Movement and Training Science, University of Wuppertal, Wuppertal, Germany; 4 Department of Intervention Research in Exercise Training, German Sport University Cologne, Cologne, Germany; 5 Institute of Occupational, Social and Environmental Medicine, Goethe University Frankfurt, Frankfurt/Main, Germany

**Keywords:** Sleep, Sleep health, Shift work, Industrial work, Work place health promotion, Interventions

## Abstract

**Background:**

In shift work, where sleeping and working is contrary to the usual circadian rhythm, poor sleep is often associated with observed higher risk for worse functioning, health, and well-being. Sleep-enhancing targeted promotion could be a promising work place health intervention to counteract these risk factors. The objective of this study was to systematically review to what extent and with what efficacy sleep interventions are already being used in shift work settings.

**Methods:**

We searched five databases (PubMed, Cochrane Library, Web of Science, Scopus, and EBSCO, until June 18th, 2024) for any controlled intervention study on shift workers adopting any sleep-enhancing interventions with any control/comparator and any outcome in the context of health, work ability, or sleep. The risk of bias tool II (for RCTs) and, for the non-randomized studies, the ROBINS-I-tool were used for risk of bias ratings.

**Results:**

The search yielded 11,261 studies after duplicate removal. Six studies (3 randomized controlled studies (RCTs), 3 non-randomized interventions, 518 participants) could finally be included in this review. Three studies adopted behavioral interventions (cognitive behavioral therapy), one used a pharmacological treatment (melatonin), and two applied an environmental approach (light exposure). Some concerns for an overall risk of bias were given in the RCTs, whilst the non-randomized controlled studies were judged to have a serious overall risk of bias. The efficacy of the interventions was mixed with beneficial and non-beneficial effects regarding the sleep parameters observed.

**Conclusions:**

In terms of optimizing industrial workers’ health, well-being and productivity, sleep interventions seem promising. Such interventions can be offered on a low-threshold without interfering everyday (working) life. Future research is needed not only to further prove the efficacy of such interventions, but also to derive practical implications and recommendations for suitable interventions.

**Trial registration:**

This systematic review was pre-registered in PROSPERO (CRD42024559360).

## Background

Shift work, a common practice in various industrial sectors [1], poses significant challenges to workers’ health and safety [2]. Approximately 20% of workers are currently engaged in shift work in industrial countries; yet this number is expected to increase [3]. Shift work refers to structured, employer-determined schedules outside typical daytime hours, such as night or rotating shifts, distinct from more flexible non-standard work [4]. The irregular and often disruptive nature of shift schedules can lead to chronic sleep deprivation, circadian rhythm disturbances, and a host of associated health problems, including cardiovascular diseases, metabolic disorders, and mental health issues [5]. Sleep disorders are common in the general working population, with prevalence estimates ranging from approximately 18% [6] to 23% [7]. An association between shift work and increasing risks of developing sleep disorders, such as insomnia, is known [8]. Furthermore, recent studies highlight that irregular sleep timing impairs cognitive performance and are linked to poorer mental health, especially on young workers [9, 10]. Disrupted sleep patterns can also compromise immune function, increasing sickness absence rates [11] Additionally, irregular sleep patterns and shift schedules pose significant challenges for shift workers in maintaining a healthy work-life balance, as they limit opportunities for family time, social interactions, and leisure activities [12]. Thus, addressing sleep disturbances in shift workers is crucial for enhancing their health, well-being and productivity [13]. In general, workplace health promotion programs cannot only improve workers’ health and productivity [14, 15], reduce absenteeism, and lower healthcare costs [16] but might also be able to impact on sleep.

Various interventions, including pharmacological treatments [17] and behavioral or environmental modifications [5], have been explored to mitigate such effects. Previous reviews on sleep disorders [18–20] and work injuries [21] suggest that workplace health programs positively impact sleep but require greater specialization for industrial workers [22, 23]. To date, however, no systematic review has quantified the impact of sleep interventions as workplace health promotion treatments for industrial workers.

Against this background, this systematic review evaluated and synthesized the current evidence on interventions designed to improve sleep parameters among industrial workers. Specifically, the review seeks to (1) identify effective strategies that stakeholders can use to enhance sleep parameters and reduce the incidence of sleep-related health issues and to (2) fill the gap of a broad range of interventions across the industrial context, thereby providing actionable insights for policymakers, employers, and healthcare providers [22, 24].

## Methods

###  Study design and search strategy

This systematic review was pre-registered in PROSPERO (CRD42024559360). An electronic literature search was conducted using the five databases: PubMed, Cochrane Library (CENTRAL including EMBASE and CINAHL), Web of Science, Scopus, and EBSCO. The search strings and strategies are listed in Table 1. The strings were adjusted for database-specific truncations, wildcards, and proximity operators. The articles were retrieved from the earliest possible date to June 18th, 2024. Preferred Reporting Items for Systematic Reviews and Meta-Analyses (PRISMA) were used to report this review [25].

**Table 1:** Search string (terms within each variable combined using AND)

Variable	Search string
Industrial workers	“industrial work*” OR factory OR manufact* OR assembly line OR blue-collar OR “construction work*”
Intervention	Intervention OR program* OR trial OR treatment OR training
Sleep	sleep

### Study eligibility criteria

Three independent investigators established the following criteria for screening titles and abstracts: randomized (RCT) and non-randomized controlled trials; articles written in English. Animal studies, reviews, and meta-analyses were excluded.

The search strings were determined using the population, intervention, comparison, and outcome scheme (PICO). Included populations were industrial workers of any gender aged 18–67 who worked at least 20 h per week in industry or manufacturing and were active for at least six months. Industrial workers were defined as those who performed manual labor during manufacturing [26]. Any intervention aiming to affect sleep parameters among workers with any passive and/or active controls and comparators, and employing any form of objective or subjective measurement, was included.

The identified studies were downloaded using a citation manager (Clarivate Analytics, EndNote 20.5, London, UK). Subsequently, the studies were transferred to Rayyan, and duplicates were removed [27]. The eligibilities of the titles and abstracts were examined before the full text was evaluated. Interventions were eligible if they explicitly targeted sleep or if sleep was assessed as a primary or secondary outcome of a broader health-related intervention. Two independent investigators (SJ, KK) executed the methodological process, and a third investigator (LR) resolved any discrepancies if necessary.

### Evaluating the risk of bias

The risk-of-bias assessment for the outcomes assessed in the RCTs was performed using the Cochrane risk-of-bias tool (ROB2) [28]. The ROB2 tool addresses biases categorized into five domains: (1) the randomization method, (2) deviations from predesignated interventions, (3) the absence of outcome data, (4) outcome measurement, and (5) selective reporting of findings. Where applicable, the ROB2 extension for crossover RCTs was utilized, which, beyond the main domains highlighted above, assesses the bias arising from the period and carryover effects [29]. An overall risk of bias domain was built based on individual judgments; all outcomes with at least one high risk for a certain bias received an overall rating of ‘high risk.’ Each domain is judged as ‘low risk of bias’, ‘some concerns’, or ‘high risk of bias’ [28].

For non-randomized studies, the ROBINS-I tool was used to judge the risk of bias [30]. The ROBINS-I tool views each study as an attempt to emulate a hypothetical pragmatic randomized trial. It assesses seven domains: (1) bias due to confounding, (2) bias due to selection of participants, (3) bias in classification of interventions, (4) bias due to deviations from intended interventions, (5) bias due to missing data, (6) bias in measurement of outcomes, and (7) bias in selection of the reported result. The judgements within each domain were ‘low risk of bias’, ‘moderate risk of bias’, ‘serious risk of bias’, ‘critical risk of bias’ or ‘No information’ [30].

Two investigators (KK, SJ) independently assessed the risk of bias of the outcomes assessed in the included studies. In cases of disagreement, a third investigator (LR) was consulted. The data were visualized with the risk of bias VISualization in R [31].

### Data extraction and evidence synthesis

One investigator (KK) retrieved the following data using the PICO scheme: (1) participants = number of participants, sex, age, and working environment; (2) intervention = type and dose (duration, time, intensity, frequency) of the intervention; (3) comparators = type and dose (duration, time, intensity, frequency) of the control/comparator group; and (4) outcomes = health-related outcomes. A second investigator (SJ) monitored, and duplicate retrieved the data process. Statistical interpretation of the results was provided only if the results were reported in the original study.

Due to the heterogeneity of outcomes and interventions of the studies, no meta-analysis could be performed. Instead, we provide a systematic narrative synthesis of the findings, using the guidance of Popay and colleagues [32].

## Results

### Literature search

**Fig. 1:**
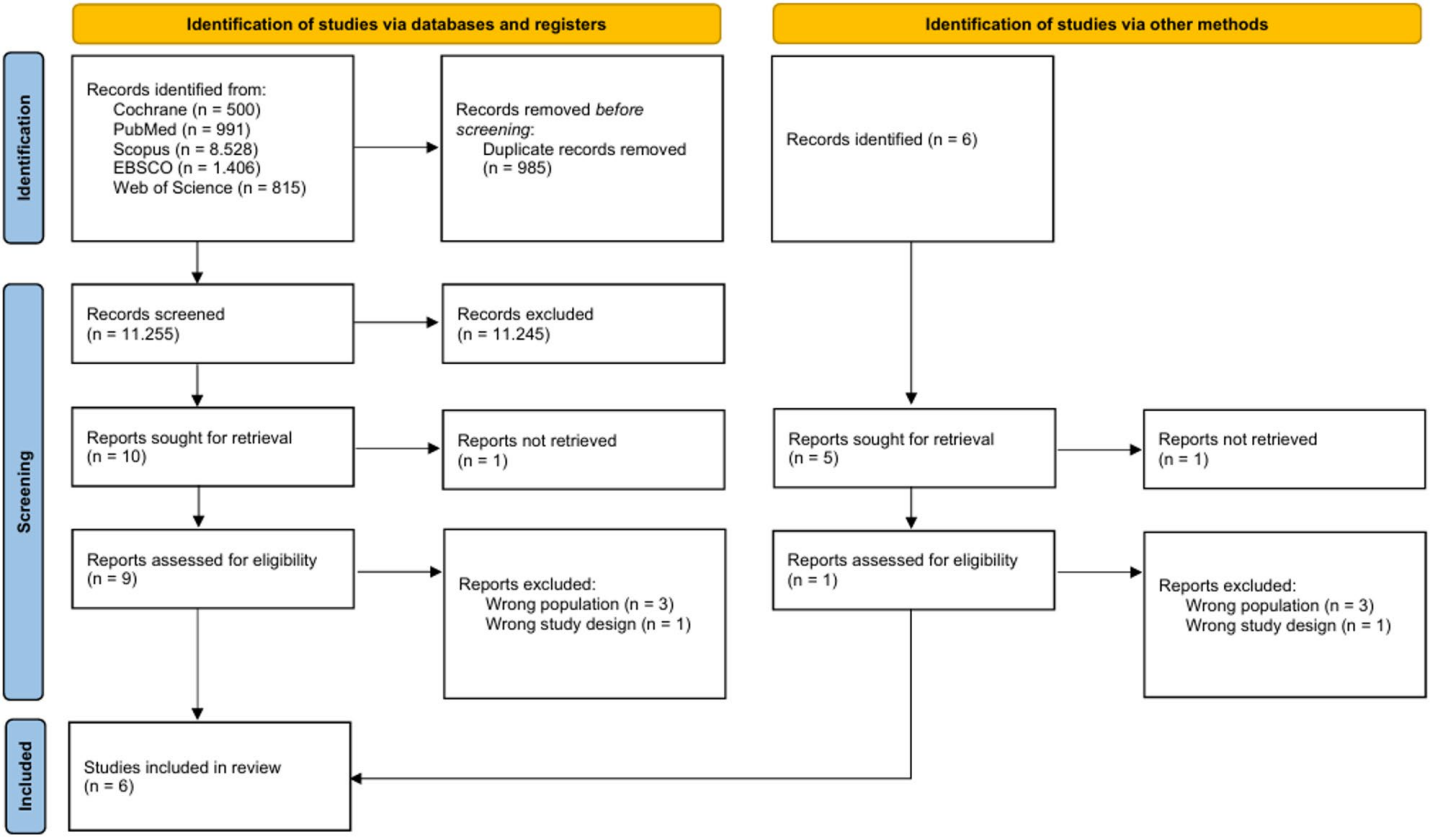
PRISMA flow diagram

Figure 1 provides the PRISMA flow diagram of the complete search process. Finally, this systematic review included overall six studies [33–38], three RCTs [35, 37, 38] and three non-RCTs [33, 34, 36].

### Description and characteristics of the included studies

The included studies are summarized in Table 2. The studies were conducted across the four countries Chile (*n* = 1 [33]), Iran (*n* = 3 [36–38]), Japan (*n* = 1 [34]), and India (*n* = 1 [35]). The publication dates ranged from 2011 to 2023.

**Table 2:** Characteristics of the studies using PICO scheme

Study	Country	Study design	Duration	Population	Intervention	Control/comparator	Outcome	Measurement tool
Barrios Araya et al. 2022 [33]	Chile	pragmatic non-randomized clinical trial with follow-up (between-subjects design; 2 groups)	2 educational sessions (90 min each; 30 days apart) and follow-up at 3 + 6 months after the intervention	Construction workers daytime with 5 construction sites (*n* = 179; study started with 2 groups of 90 workers; 85.6% male (82.2% in the control group and 88.9% in the intervention group); mean age: 44.19 ± 12.26 years)	2 × 1.5 h sleep education (subdivision of the intervention group into 3 groups of 30 each) in the workplace + handout of educational material and follow-up activities to be implemented daily for 30 days	wait-list (received information on sleep hygiene at the end of the study)	sleep quality, sleepiness, fatigue	Pittsburgh Sleep Quality Index, Epworth Sleepiness Scale, PCPVT fatigue test
Karchani et al. 2011 [38]	Iran	2 × 2 cross-over (within-subjects design; 2 groups)	2 periods of 2 nights and washout of 6 days (normal schedule: 2 days on morning shift, 2 days on evening shift, 2 days off work, 2 days on night shift)	Shift workers at a metallurgy production plant (*n* = 90; 100% male; mean age: 30 years (range 30–36 years))	exposure to bright light (2500–3000 lx) during 4 15-minute breaks at night shifts (2 h interval between the 4 breaks, with the first exposure starting at 22:00 h)	normal light (300 lx)	sleepiness	Stanford Sleepiness Scale
Kubo et al. 2011 [34]	Japan	2 × 2 cross-over (within-subjects design; 2 groups)	3 consecutive weeks (1 week baseline, 1 week intervention, 1 week control)	Manufacturing workers with habitually short sleep periods (*n* = 28; 18 men and 10 women; mean age: 38.3 ± 8.1 years; < 6 h mean sleep duration on weekdays)	stay in bed for ≥ 8 h between 22:00–09:00 h on weekends (Friday to Sunday; no daytime naps allowed)	habitual weekend sleep-wake patterns	psychomotor vigilance, self-rated fatigue symptoms, blood pressure, total sleep time (TST), sleep efficiency (SE), bedtime, wake time	Psychomotor Vigilance Task, fatigue questionnaire, blood pressure, Micro-mini RR actigraph
Pravalika et al. 2023 [35]	India	parallel randomized controlled open-label trial (between-subjects design; 2 groups)	8 consecutive weeks (daily 1 h intervention)	Manufacturing workers (31% shift workers) with self-reported musculoskeletal pain and discomfort in machinery industries (*n* = 90; 100% male; mean age: 40.57 ± 6.85 years (range 18–60 years))	8 weeks yoga intervention (à 60 min/day for 5 days/week) in the workplace	wait-list (received lifestyle suggestions)	musculoskeletal pain and discomfort (primary outcome); perceived stress sleep quality (secondary outcome)	Cornell Musculoskeletal Discomfort Questionnaire, Visual Analogue Scale, Perceived Stress Scale, Pittsburgh Sleep Quality Index
Sadeghniiat-Haghighi et al. 2016 [37]	Iran	randomized, double-blind, placebo-controlled crossover study (within-subjects design; 2 groups)	3 periods of 3 nights and washouts of 2 weeks (3 nights baseline, 3 nights intervention, 3 nights control; rotation shift: 1 week day shift followed by 1 week night shift without days off)	Shift workers from a Refinery Oil company with difficulty falling asleep (*n* = 39; study started with 2 groups of 25 workers; 100% male; mean age: 32.9 ± 8 years (range 24–52 years))	3 mg tablet of melatonin about 30 min before usual bedtime	placebo (identical in shape and color)	total sleep time (TST), sleep onset latency (SOL), sleep efficiency (SE), wakening after sleep onset (WASO)	Insomnia Severity Index, Somnowatch
Sadeghniiat-Haghighi et al. 2011 [36]	Iran	2 × 2 cross-over (within-subjects design; 2 groups)	2 periods of 2 nights and washout of 4 days (2 12-hour day shifts followed by 2 days off work, and 2 12-hour night shifts)	Shift workers at a ceramic factory (*n* = 94; 100% male; mean age: 33 years (range 21–45 years))	exposure to bright light (2500 lx) during 2 20-minute breaks at night shifts (2 h interval between the 2 breaks, with the first break starting at 00:30 h)	normal light (300 lx)	sleepiness	Stanford Sleepiness Scale

The investigated population consisted of manufacturing shift workers (*n* = 3 [34, 35, 38]), shift workers (*n* = 2 [36, 37]), and construction shift workers (*n* = 1 [33]). The age of the participants ranged from 18 to 65 years, with samples ranging from 26 [34] to 179 [33] (mean 86; median 90), representing a total of 518 industrial workers, with 445 investigated males, 34 females, and 39 workers without an exact designation of sex. Three studies investigated men only [35, 36, 38]. One study used a cognitive-behavioral intervention lasting one month [33], one study used a three day melatonin therapy [37], one study used an eight week yoga program with additional lifestyle advice [35], one study used a three-week duration sleep extension on weekends [34], and two studies used six [36] respectively four days [38] of different light exposures as an environmental modification. One study [33] reported a six-month follow-up period.

The control groups in these studies differed largely, from placebo [37], information about sleep hygiene [33], lifestyle advice [35], to true control [34, 36].

Various outcome measures to assess sleep were used. Four studies used only self-reported measures [33, 35, 36, 38], one study used only objective measurements [37], and one study used both objective and self-reported measurements [34] (Table 2).

### Risk of bias

**Fig. 2:**
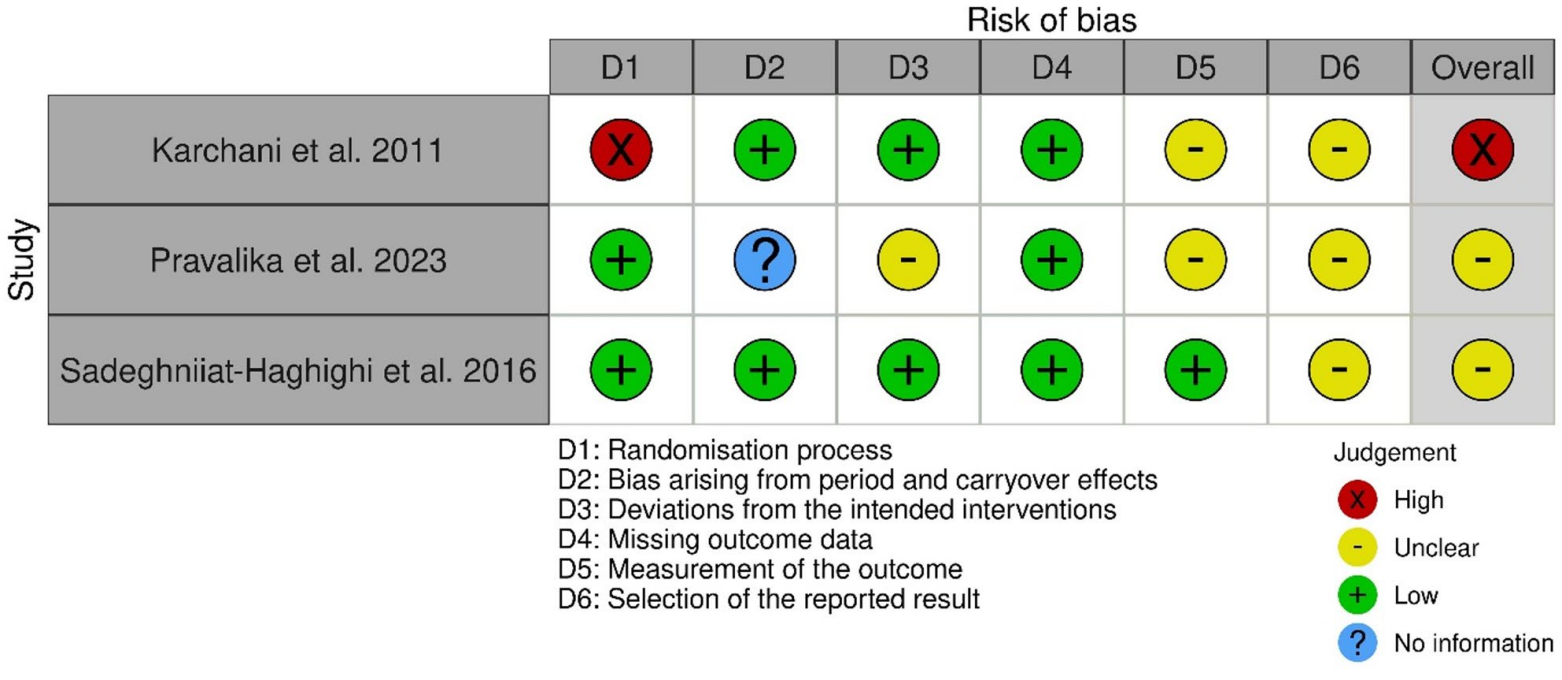
Traffic light plot for the individual risk of bias domains for the included randomized controlled trials

**Fig. 3:**
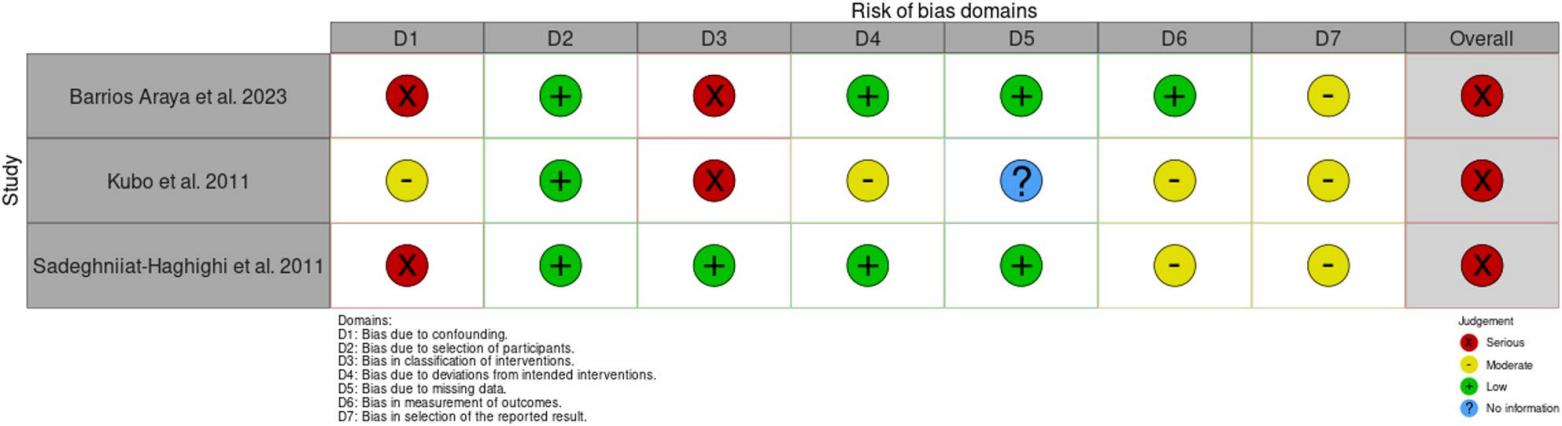
Traffic light plot for the individual risk of bias domains for the included non-randomized controlled trials

The results of the risk of bias assessments are illustrated in Figs. 2 and 3 . One RCT was rated as having a “high” overall risk of bias [38], primarily due to concerns related to the randomization process. The other two RCTs were judged to have some concerns” for overall risk of bias of [35, 37], mainly due to potential bias in the selection of reported results. Except for the domain selection of reported results, one RCT assessed only a low risk of bias [37]. In the RCT by Pravalika and colleagues [35], other reasons for risk of bias of some concerns were deviations from the intended interventions, measurement of the outcome, and selection of the reported result. None of the included RCTs were judged to have either a low or high risk of bias.

All non-randomized studies were judged to have a serious overall risk of bias [33, 34, 36]; mainly because of potential bias due to confounding and bias in the classification of interventions. The possible bias from selecting participants was assessed as low risk in all studies, and the bias from missing data was deemed low risk in two out of the three studies, except for one study where no information was available. For the domain Bias in selecting the reported result, all studies were judged with a moderate risk of bias. None of the studies was judged to have a low risk of bias.

### Effectiveness of studies included on sleep parameters

#### Interventions- pharmacological treatment

One double-blind, placebo-controlled, crossover RCT aimed to evaluate the efficacy of 3 mg melatonin therapy in a refinery oil company shift workers. The study showed significant improvements in sleep onset latency (treatment: 0.20 ± 0.15 h vs. placebo: 0.31 ± 0.16 h) and sleep efficiency (treatment: 85.5 ± 6.3% vs. placebo: 82.54 ± 8.1%), but not in total sleep time (treatment: 5.9 ± 0.97 h vs. placebo: 5.6 ± 0.97 h) and waking after sleep onset (treatment: 0.81 ± 0.39 h vs. placebo: 0.9 ± 0.52 h), compared to the control group on average of three days [37].

#### Environmental

Two studies have implemented environmental change as a treatment for sleep among industrial workers. In those crossover studies, male shift workers were exposed to bright or normal light in their night shifts. One study conducted the light conditions on two 20-minute breaks. The results indicated significantly reduced sleepiness ratings with bright light exposure during both break periods (break 1: treatment 2.43 vs. control 2.77; break 2: treatment 2.07 vs. control 3.07) [36]. The other RCT permitted four 15-minute breaks and assessed the subjects’ sleepiness one hour after the light exposure. Here, too, the results showed a significant treatment effect for the bright light exposure (total mean first night treatment 2.533 vs. control 3.144) [38].

#### Behavioral

Three studies that adopted behavioral approaches in workplace health promotion programs for industrial workers were identified [33–35]. One non-RCT investigated whether extending sleep duration during weekends improves sleep efficiency, psychomotor vigilance, reaction times, subjective fatigue, and blood pressure. Manufacturing workers with weekday sleep durations of less than six hours per night were instructed to stay in bed for more than eight hours per night over three weeks. The results indicated no significant improvement in any of the outcomes. However, a significantly shorter reaction time was observed at the beginning of the week. In contrast, significantly longer reaction times were noted at the end of the week in the waiting-list control group [34]. Another non-RCT used a cognitive behavioral intervention on construction workers to reduce fatigue by improving sleep quality over one month. Two sessions were conducted over one month. They showed non-significant improvements in any parameter post-intervention, but a significant improvement in reducing sleepiness, measured with the Epworth sleepiness scale (ESS; treatment 6.56 ± 4.53 vs. control 9.32 ± 5.75), and fatigue, measured via reaction time (PCPVT; treatment 309.46 ± 60.04 vs. control 303.36 ± 81.44) in the post-intervention follow-up of six months compared to the control group [33], who received only information about sleep hygiene. A randomized controlled trial over eight weeks used a yoga program and additional lifestyle advice from a machinery manufacturing company on male industrial workers. A significant beneficial difference was detected for musculoskeletal pain and discomfort as measured by the Cornell musculoskeletal discomfort questionnaire (treatment 1.861 ± 3.771 vs. control 3.271 ± 4.791), pain assessed via VAS-scale (treatment 0.654 ± 0.793 vs. control 0.969 ± 1.008), stress through the perceived stress scale (treatment 9.422 ± 6.781 vs. control 12.222 ± 5.885), and the Pittsburgh sleep quality index (treatment 3.111 ± 2.656 vs. control 4.667 ± 4.194) compared to a control group with lifestyle advice regarding food and sleep [35].

## Discussion

In total, six studies were included in this systematic review on the efficacy of interventions to improve sleep parameters among industrial workers. Among these six studies, three adopted behavioral interventions, one used a pharmacological treatment approach, and two used an environmental approach. Three included studies were randomized controlled studies; the other three were non-randomized controlled studies. The randomized controlled studies had a risk of bias with some concerns. In contrast, the non-randomized controlled studies were judged to have a serious risk of bias, necessitating a cautious interpretation of all results. The efficacy of the interventions was mixed with beneficial and non-beneficial effects regarding the sleep parameters observed.

### Intervention characteristics

The measurement approaches used in the included studies varied considerably, with three studies relying exclusively on self-reported measures, one utilizing both self-reported and objective methods, and one, after applying the Pittsburgh Sleep Quality Index (PSQI) as a screening tool, employed only objective measurements. The PSQI was the most commonly used tool; it displays good reliability and validity [39]. Subjective tools like questionnaires and sleep diaries are cost-effective and capture personal sleep experiences but are prone to recall bias and inaccuracies [40, 41]. In contrast, objective methods provide precise physiological data but are often costly, invasive, and may lack ecological validity. Integrating both approaches allows for a more comprehensive and balanced evaluation of sleep by combining experiential insights with biological precision [41, 42]. Therefore, to enhance methodological rigor and validity, future research should prioritize integrating objective measures, which may also include parameters derived from industry-specific data sources [43, 44], to provide a more comprehensive evaluation of sleep outcomes in industrial worker populations.

The duration of interventions in the included studies varied widely, ranging from two sessions over 30 days to interventions lasting up to 8 weeks. Some studies implemented shorter durations, including four days to two-week washout periods. However, shorter durations may be insufficient, as changes depend on the frequency and direction of the modifications individuals consider [45]. Additionally, only one of the included studies had a true post-intervention follow-up, which should be considered. In other studies, particularly those using crossover designs, follow-up assessments were not feasible or not reported. The variability highlights the need for future research to establish the most effective intervention length promotion of sleep among industrial workers.

Notably, none of the included studies implemented work-related outcomes such as productivity or sickness absence. Future research on workplace interventions should incorporate these measures to evaluate the potential impact on work-related outcomes through workplace health promotion [46].

### Effectiveness on sleep outcomes

Only one study used a pharmacological approach [37], which is somewhat surprising because pharmacological treatments for insomnia are widely used in practice [47]. In this study of shift workers, a 3 mg melatonin therapy with a duration of three nights was found to improve sleep onset and efficiency. However, it did not significantly alter total sleep time or waking after sleep onset compared to the control group [37]. These findings partially contradict the results of meta-analyses, which generally indicate that melatonin has no significant effect on improving sleep onset latency, total sleep time, or sleep efficiency in adults [48]. However, another meta-analysis highlighted significant improvements in melatonin levels related to sleep onset latency and total sleep time for individuals with secondary sleep disorders, such as shift work. Still, the data revealed no impact on sleep efficiency [49]. According to American Academy of Sleep Medicine (AASM) guidelines, melatonin is generally not recommended as first-line treatment for primary insomnia [50]. Nonetheless, pharmacological treatments for sleep disorders, which vary based on their mechanisms of action and affect different sleep parameters, have distinct safety risks. However, their long-term effects remain unclear [51]. Our review yielded only one study that involved melatonin administration or other pharmacological treatments. In addition, the number of studies included in the meta-analysis was limited. Therefore, the results must be interpreted cautiously, leaving room for positive effects, especially clinically relevant differences. Pharmacotherapies often have a short duration of action and can cause a hangover effect [52], indicating that a longer duration might be necessary to observe beneficial effects. Future studies on pharmacological treatments for industrial workers should also include a follow-up to assess the potential hangover effects.

Only two studies utilized an environmental approach through exposure to light [36, 38]. Existing research suggests that environmental factors such as noise [53] and light [54] significantly influence sleep. Light, in particular, is crucial in regulating the circadian rhythm and addressing sleep disturbances, such as insomnia [55]. Given this, it is surprising that more studies have yet to explore this approach, especially considering the existing recommendations to incorporate environmental and organizational strategies to improve health outcomes [56]. A recent review examining evening bright light exposure in shift workers reported favorable outcomes, including circadian phase delays, improved sleep, and enhanced performance without adverse effects [54]. Similarly, a review focusing on night-shift nurses found that bright-light interventions reduced sleepiness and insomnia [57]. The effect of light therapy on sleep was also deemed favorable [13]. A systematic review, however, noted that bright light during the night of a shift revealed a reduction in sleepiness, but with a very limited number of studies and low quality [58]. These findings align with our review’s results, emphasizing and highlighting the need for further research. These findings underscore the potential benefits of light-based interventions and highlight the research gap in applying this approach across a broader industrial workforce.

In addition to the other interventions, behavioral approaches to workplace health promotion have gained increasing attention, particularly for their potential to address sleep disturbances among industrial workers. A systematic review highlighted the effectiveness of non-pharmacological interventions on sleep disturbances [59].

Our review identified a single RCT that employed an eight-week yoga program combined with lifestyle advice, demonstrating improved pain, stress, and sleep quality [35]. These findings are consistent with systematic reviews and meta-analyses that emphasize the benefits of yoga in sleep management [60–62]. Yoga, an ancient practice involving postures, controlled breathing, and meditation [63], has shown positive effects on physical and mental health, particularly in reducing depressive symptoms, without reported adverse effects, which likely contributes to its impact on stress and sleep quality [64]. Yoga has also been shown to improve outcomes for specific clinical populations, such as individuals with type 2 diabetes [65] and rheumatic diseases [66], conditions that are particularly relevant to industrial workers [67, 68]. Regular exercise, including yoga, regulates circadian rhythm and positively impacts sleep quality [61, 69]. The European guideline for diagnosing and treating insomnia suggests that exercise can effectively manage insomnia [70]. Moreover, network meta-analyses have identified yoga as highly effective for improving sleep quality [71, 72]. However, the intensity of the exercise is crucial in determining its effectiveness in enhancing sleep quality. A network meta-analysis indicates that moderate-intensity muscle endurance training combined with walking is the best treatment for insomnia, followed by yoga if cognitive behavioral therapy is not an option [62]. Besides the low number of findings in our review, the literature underscores the potential of yoga as a holistic and practical strategy for workplace health promotion in industrial settings.

Psychosocial interventions, including cognitive behavior therapy, have demonstrated small to moderate effects on mental health, as reported in an umbrella review by Miguel and colleagues [73]. In our review, we identified a non-RCT that evaluated cognitive behavioral therapy, which showed that workers who received this intervention experienced reduced sleepiness and fatigue [33], underscoring its potential utility in workplace settings. The efficacy of cognitive behavioral therapy is well-established across various populations [13, 62, 70, 74], and additional findings suggest its promise for improving presenteeism [75]. However, the complexity of cognitive behavioral therapies can present challenges for adherence [52]. Emerging digital cognitive behavioral therapy interventions may offer solutions to improving accessibility and supporting outcomes related to mental health, sleep, and productivity [76, 77]. Nevertheless, further high-quality randomized controlled trials are necessary to strengthen the evidence base for cognitive behavioral therapy among industrial workers.

Conversely, a non-RCT found that extending weekend sleep duration did not improve sleep efficiency or other health parameters [34]. These findings align with a study that reported no significant difference between individuals who remained sleep-deprived over the weekend and those who attempted to catch up on sleep [78]. Chronic sleep deprivation, defined as sleeping fewer than seven hours per night, is linked to an increased risk of cardiovascular disease, obesity, diabetes, hypertension, depression, and higher overall mortality [23]. Insufficient sleep also induces behavioral changes, such as nutritional intake, which may exacerbate health risks [79]. Shift work further complicates these challenges, as meta-analysis indicated that shift workers experience worse sleep quality despite achieving longer total sleep durations than day shift workers [80]. These sleep disruptions can impair cognitive function and reaction times, reduce workplace safety and productivity, increase sickness absence, and reduce job satisfaction [18]. Prevention may be the most effective approach to address these issues. While recovering sleep debt is difficult, adopting behavioral changes such as sleep habits through education may enhance sleep quality [81].

### Risk of bias

This overall risk of bias was lower than other reviews on industrial workers, which frequently report a high risk of bias. For the RCTs, the primary concern was the selection of the reported result, leading to an unclear risk of bias. This type of bias can result in reporting bias, potentially overestimating or underestimating the true effects of the intervention [82]. Aside from this domain, one RCT was assessed as having an overall low risk of bias. At the same time, the other demonstrated additional unclear risks related to deviations from intended interventions and outcome measurements.

In contrast, all non-randomized studies were judged to have an overall risk of serious bias. The main reasons for this were bias due to confounding factors and bias in the classification of interventions. Both biases can misestimate intervention effects or misclassify participants, reducing the validity of study findings [83].

### Population- and workplace-specific needs

In the industrial sector, it is crucial to consider the impact of shift [84] alongside sex-specific needs and health hazards [85]. Most industrial workers in this review included were male, indicating a potential need for interventions tailored to this group [86]. However, it is also important to note that women are disproportionally affected by sleep disorders, with insomnia being approximately 1.4 times more common in women than in men [60]. Additionally, gender-specific differences in how shift work affects sleep have been observed, particularly regarding metabolic syndrome [3]. Although some studies addressed insomnia-like symptoms, none required a formal sleep disorder diagnosis, limiting generalizability to clinically diagnosed populations. Sleep also acts as a catalyst for other health-related outcomes and should be a key focus [87].

The wide range of industrial tasks [88] and work environments [89] further affect sleep-related factors. These factors are also shaped by individual characteristics such as age [80], stress [90, 91], and BMI [75]. Findings from an umbrella review further emphasize the critical relationship between sleep, health, and shift work, reinforcing the importance of these considerations [92]. The varying health conditions among workers underscore the need for interventions that address their distinct characteristics [93–95]. Tailored interventions are particularly crucial in low-income countries, where resources and working conditions often differ significantly [96].

### Strengths and limitations of this review

This review comprehensively analyzes workplace health promotion interventions targeting sleep parameters among industrial workers. By focusing on a specific population, the review offers valuable insights, highlighting both interventions’ beneficial and non-beneficial effects on sleep outcomes.

However, this review has limitations. The small number of included studies restricts the generalizability of the findings, while the high heterogeneity of interventions complicates direct comparisons and synthesis. As a result, narrative synthesis was employed to address this variability. Additionally, only published studies were included, introducing the possibility of publication bias, which may impact the findings. These limitations should be considered when interpreting the findings and their implications for workplace health promotion.

### Recommendations for future research and practical applications

This review provides valuable insights into workplace health promotion and offers a foundation for practical applications to improve sleep health among industrial workers. Integrating sleep health into workplace health promotion should be prioritized, focusing on wearable devices and mobile applications to monitor sleep and health-related metrics, enhance ergonomics and safety, and promote overall well-being [97, 98]. E-health platforms present a promising approach to delivering accessible, evidence-based interventions that accommodate the irregular schedules of shift workers [73].

Future research should explore the feasibility and acceptance of technological interventions to enhance workplace health promotion. Efforts should also focus on maintaining healthy circadian rhythms, vital for mental health and well-being [99], and addressing the role of shift changes and occupational demands in perpetuating insomnia [100]. To ensure accurate and meaningful outcomes, future studies should include only valid and reliable measures of sleep, ideally integrating both subjective and objective tools and meeting established standards [101]. Additionally, potential cofounders, such as work schedules and leisure activities, should be measured to provide a more comprehensive understanding of sleep health influences on industrial workers. Furthermore, to sleep parameters, work-related outcomes such as presenteeism should be incorporated into the evaluation of workplace interventions [75]. Long-term follow-up studies are essential to assess the sustainability of intervention benefits, as are systematic and well-designed workplace interventions on industrial workers. Effective organizational interventions, such as optimizing shift scheduling and rotation, should be implemented and evaluated like for other health issues among shift work [46].

## Conclusions

By providing a comprehensive overview of existing workplace interventions and their impact on sleep parameters, this review highlights the potential benefits of workplace health promotion for industrial workers. However, the limited number of included studies, along with concerns about study quality due to risk of bias, presents challenges in drawing robust conclusions.

The heterogeneity of interventions complicates the formulation of generalizable conclusions, emphasizing the need for future research to identify the specific elements that enhance efficacy and adoption in workplace settings. To tailor interventions effectively, the distinct health characteristics and conditions of industrial workers must also be addressed.

Despite these limitations, the positive outcomes reported in some studies suggest a promising role for workplace interventions in improving industrial workers’ sleep health. These findings support the importance of integrating sleep health into workplace health promotion programs and advancing public health at national and global levels to improve the health and well-being of industrial workers.

## Data Availability

No datasets were generated or analysed during the current study.
